# Lithium doped biphasic calcium phosphate: Structural analysis and osteo/odontogenic potential *in vitro*

**DOI:** 10.3389/fbioe.2022.993126

**Published:** 2022-11-08

**Authors:** Kyung-Hyeon Yoo, Yeon Kim, Yong-Il Kim, Moon-Kyoung Bae, Seog-Young Yoon

**Affiliations:** ^1^ School of Materials Science and Engineering, Pusan National University, Busan, South Korea; ^2^ Department of Oral Physiology, School of Dentistry, Pusan National University, Yangsan, South Korea; ^3^ Department of Orthodontics, Dental Research Institute, Pusan National University, Yangsan, South Korea

**Keywords:** biphasic calcium phosphate, lithium doping, crystal structure, characterization, osteogenesis

## Abstract

Biphasic calcium phosphate (BCP) is generally considered a good synthetic bone graft material with osteoinductive potential. Lithium ions are trace elements that play a role in the bone-remodeling process. This study aimed to investigate the effects of lithium ions on the phase, crystal structure, and biological responses of lithium doped BCPs and to identify improvements in their osteogenic properties. Lithium-doped BCP powders with different doping levels (0, 5, 10, and 20 at%) were synthesized *via* the co-precipitation method. We found that the four types of lithium-doped BCP powders showed different phase compositions of hydroxyapatite and β-tricalcium phosphate. In addition, lithium ions favored entering the β-tricalcium phosphate structure at the Ca (4) sites and calcium vacancy sites [V_Ca_(4)] up to 10 at%. This substitution improves the crystal stabilization by filling the vacancies with Ca^2+^ and Li^+^ in all Ca sites. However, when the concentration of Li ions was higher than 10 at%, lithium-induced crystal instability resulted in the burst release of lithium ions, and the osteogenic behavior of human dental pulp stem cells did not improve further. Although lithium ions regulate osteogenic properties, it is important to determine the optimal amount of lithium in BCPs. In this study, the most effective lithium doping level in BCP was approximately 10 at% to improve its biological properties and facilitate medical applications.

## 1 Introduction

Millions of patients suffering from bone defects caused by various causes, such as fractures, diseases, and tumors, require bone grafting treatments to repair these defects (Basu and Basu, 2019; Bohner et al., 2020). To reconstruct hard tissue, both biological (autograft, allograft, xenograft) and synthetic (ceramics) materials are used in clinical settings (Bauer and Muschler, 2000; Campana et al., 2014; Basu and Ghosh, 2017). Because of the limited availability of biological bone substitutes, various studies on synthetic bone substitution materials have been conducted to enhance their clinical use. Calcium phosphate materials, one of the most widely employed synthetic ceramics, are well known for their good biocompatibility, osteoconductivity, and biofunctions in cells. They are classified according to the Ca/P ratio, such as hydroxyapatite (HAp; 1.67), β-tricalcium phosphate (β-TCP; 1.5), and their mixture of biphasic calcium phosphate (BCP; 1.5–1.67). They can also be used as bone graft substitutes in dental and orthopedic reconstructive medicine (Hench and Wilson, 1984; Cerroni et al., 2002; Padilla et al., 2005). BCP is generally considered to be a moderate biomaterial with the advantages of both HAp and β-TCP and is expected to show osteoinductive potential without any growth factors (Kim et al., 2012a; Samavedi et al., 2013). The phase compositions of HAp and β-TCP can affect the overall properties of BCP, especially by controlling new bone formation (Arinzeh et al., 2005; Dorozhkin, 2015). However, they cannot effectively develop osteogenic behavior to achieve optimal conditions for bone grafting.

To improve these biological properties, a method for stimulating the osteogenic pathway is required. Over the past several years, numerous trace elements related to bone remodeling, such as strontium (Boanini et al., 2011; Marques et al., 2016), silicon (Pietak et al., 2007), magnesium (Ryu et al., 2004; Frasnelli and Sglavo, 2016), zinc (Luo et al., 2014; Lu et al., 2021), sodium (Yoshida et al., 2006; Boanini et al., 2010), potassium (Kannan et al., 2007; Matsumoto et al., 2009), and lithium (Vahabzadeh et al., 2017), have been substituted with calcium phosphate materials. Despite attempts to effectively improve the various properties of calcium phosphate, this improvement was only effective in a specific ion content range (Singh et al., 2016; Cho and Kwun, 2018). There are substitution limits in calcium phosphate, with recommended doping ion content. When the amount of substituted ions is less than the appropriate amount, the doped ions do not affect the overall bone-formation process because they are too small to stimulate the osteogenic pathway (Luo et al., 2014; Singh et al., 2016). Excess accumulation of doping ions causes various side effects, such as cytotoxicity and inhibition of biological activity (Wätjen et al., 2002). Therefore, the optimal doping content depends on the types of trace elements and base materials, and it is important to study ion-doped calcium phosphates to improve their biological performances.

Lithium is used as an antidepressant and for the treatment of bipolar disorders (Young, 2009). Many studies have investigated the effects of lithium (Li) on bone regeneration and reported that Li stimulates the Wnt signaling pathway, which has a crucial role in the bone remodeling process (Klein and Melton, 1996; Rao et al., 2005; Wang et al., 2016; Huang et al., 2019). Bioglass and calcium phosphate coatings with lithium ions have also been reported (Han et al., 2012; Thorfve et al., 2014; Zhang et al., 2019). According to Han et al. (Han et al., 2014), Li-containing β-TCP stimulates osteogenic gene expression in both human periodontal ligament and human bone marrow stromal cells. However, the toxicity of lithium ions may be observed at higher concentrations (1.5 mM in the serum) and should be monitored carefully (Timmer and Sands, 1999; Young, 2009). In the dental area, human dental pulp stem cells (hDPSCs) play an important role in osteo/odontogenesis (Mortada and Mortada, 2018). Therefore, it is important to study the effect of substituted lithium ions in BCP powder on the osteo/odontogenic behavior of hDPSCs.

We hypothesized that the optimal level of lithium will maximize the biological properties of BCP, especially its osteogenic properties. To validate our hypothesis, Li-BCP powders with different amounts of lithium were synthesized using a co-precipitation method. Then, the effects of lithium content on the phase composition and structure variation of Li-BCP were investigated. To identify its osteogenic responses, the behavior of hDPSCs with Li-BCP was examined *via* the3-(4,5-dimethyl thiazol-2-yl)-2,5-diphenyltetrazolium bromide (MTT) assay, alkaline Phosphatase (ALP) staining, and Alizarin Red S (ARS) staining.

## 2 Materials and methods

### 2.1 Powder preparation

BCP and Li-BCP were synthesized using a chemical co-precipitation method. Calcium nitrate tetrahydrate (Ca (NO_3_)_2_•4H_2_O, Junsei >98%), diammonium hydrogen phosphate ((NH_4_)_2_•HPO_4_, Junsei >99%), and lithium nitrate (LiNO_3_, Junsei >99%) were used as the Ca, P, Li ions precursors, respectively. To synthesize pure BCP powder, an appropriate amount of calcium nitrate tetrahydrate was dissolved in deionized (D.I.) water by stirring at 45°C for 30 min (solution 1). Diammonium hydrogen phosphate solution was slowly added (dropwise, 13 ml/min) to the calcium nitrate tetrahydrate solution to achieve a predetermined Ca/P molar ratio of 1.602, providing a HAp:β-TCP ratio is 60:40 in BCP (Kim et al., 2012b). Li-doped BCP powders were synthesized with different amounts of lithium (0, 5, 10, 20 at%). Calcium nitrate tetrahydrate and lithium nitrate were dissolved in D.I. water (solution 1′), and diammonium hydrogen phosphate was dissolved separately in D.I. water. Both solutions were stirred at 40°C for 30 min, and then solution 1′ was slowly added dropwise (13 ml/min) to the other solution. In both pure BCP and Li-BCP co-precipitation processes, the pH of the reaction solutions was maintained at 11 using the ammonium hydroxide (NH_4_OH, Junsei) solution. Additional mixing was performed for 2 h after complete pouring, and the solution was aged at 40°C in the water bath. After 24 h, the precipitate was filtered and dried at 80°C for 24 h in a drying oven. The dried cake was crushed to obtain a fine powder. Subsequently, each powder was calcined at 1,000°C for 2 h (at a heating rate of 6°C/min with natural cooling) in air.

### 2.2 Comprehensive structural analysis of Li-BCP

The composition of synthesized powder was quantified using an inductively coupled plasma atomic emission spectrometer (ICP-OES; ACTIVA, JY HORIBA, France) with argon plasma at 6000 K at the Korea Basic Science Institute. The collected media (extracts) were also analyzed to determine the concentrations of calcium and lithium using ICP-OES in the ppm range. The phases and crystal structures of the calcined powders were investigated *via* X-ray diffraction (XRD, XPERT-3; Malvern Panalytical, United Kingdom) using Cu Kα radiation (l = 1.5418 Å, 40 kV, 30 mA). Data were collected for 2θ ranging from 10° to 70°, with step size and duration time of 0.01 and 70 s, respectively. To analyze the phase of the synthesized powders, we used the integrated X-ray powder diffraction software package PDXL (Rigaku, Version 2.1.3.4) using ICSD card no. 97500 (β-TCP) and no. 183744 (HAp). Crystal structure analysis of the powder was carried out by Rietveld refinement using Fullprof software to obtain the crystallographic information file (CIF). Morphology of the calcined powder were conducted using a field emission scanning electron microscope (FE-SEM; MIRA3, TESCAN, Männedorf, Switzerland). The bonding structures were obtained using a Raman Spectrometer (UniRAM; UniNano Tech, Korea) with laser light at a wavelength of 532 nm. The phosphate and hydroxyl bonds were recorded in the wavenumber ranges of 200–1,600 cm^−1^ and 3,500–3,650 cm^−1^, respectively. Various functional groups in the Li-BCP samples were identified using attenuated total reflection-Fourier-transform infrared spectroscopy (IS50; Thermo Fisher Scientific, Waltham, MA, United States). The spectra were recorded from 2000 to 400 cm^−1^ with 32 scans at a resolution of 4 cm^−1^.

### 2.3 Preparation of extracts

To understand the initial dissolution behavior, all powders were extracted from the culture medium, according to the indirect method proposed by the International Standard Organization (ISO 10993–12) (Standardization, 2012). Depending on the standard, the powder was immersed in a minimum essential medium (α-MEM) with 10% fetal bovine serum (10% FBS, pH = 7.4) at 0.1 g/ml concentration. Before the test, each powder was sieved and got the same range of particle size (45–75 μm). After 24 h, the aged mixtures (incubated at 37°C) were centrifuged, and extracts were collected using a 0.20 μm pore-size syringe filter. Calcium and lithium-ion concentrations in the collected media were analyzed using ICP-OES in the ppm range.

### 2.4 Cytotoxicity test

For the cell cytotoxicity test, hDPSCs purchased from Lonza (PT-5025; Basel, Switzerland) at passage 8 were used. The cytotoxicity of the synthesized powder was evaluated using the MTT assay (Ahn et al., 2020). To determine the proper concentration of powder, a pre-test was conducted with four different amounts of powder (125, 250, 500, and 1,000 μg/well). hDPSCs were seeded at a density of 1 × 10^4^ cells/well in a 24-well culture plate. The cells were cultured in the material extraction full media-containing α-MEM (Gibco), 10% FBS (Merck), 1% penicillin-streptomycin (Gibco), and 5 μg/ml plasmocin (InvivoGen, San Diego, CA) in a 5% CO_2_ incubator at 37°C for 24 h. The culture medium in each well was replaced with 100 μL fresh culture medium containing 10 μL MTT solution (5 mg/ml MTT in sterile phosphate-buffered saline; Sigma-Aldrich, St. Louis, MO, United States) and incubated for 4 h at 37°C and 5% CO_2_. The medium was replaced with 100 μL of dimethyl sulfoxide (Sigma-Aldrich, St. Louis, MO, United States) for 5 min at 37°C. The absorbance was subsequently measured at 570 nm using a microplate reader (Dynex, Lincoln, United Kingdom). After determining the proper powder concentration, the MTT assay was conducted using the same process for 1, 2, 3, and 7 days. All results were obtained from three independent experiments and control wells were also prepared with full media.

### 2.5 Osteo/odontogenic differentiation behavior

To identify the effect of lithium on the BCP and degraded lithium ions on hDPSCs, osteogenic differentiation tests were conducted using two different processes. The first sample preparation was a direct method using 1 mg/well of the synthesized powder. This method indicated the effect of the crystal structure on the biological properties of the synthesized powder. The second process involved extraction using the extracted powder after immersion in the cell culture medium (0.1 g/ml) according to ISO 10993–12 (Standardization, 2012). Using this extraction method, we identified the effect of ions degraded from the synthesized powder on the cell behavior.

#### 2.5.1 ALP staining

hDPSCs were cultured at 2 × 10^4^ cells in a 48-well plate and incubated for 1 day before treatment. After 1 day, the medium was replaced with an osteogenic differentiation medium (ODM) containing 100 nM ascorbic acid (Sigma-Aldrich), 10 mM β-glycerophosphate (Sigma-Aldrich), and 100 nM dexamethasone (Sigma-Aldrich) and incubated for 7 days. In this experiment, osteogenic medium was used as positive control, while the full media was used as negative control. The osteogenic medium was changed every two or 3 days. After 7 days, osteogenic differentiation was observed using an ALP staining kit (86R-1KT; Sigma-Aldrich, United States). Staining images were quantified using an inverted microscope equipped with a digital camera (Optinity KCS3-2000SS; Korea Lab Tech, Korea), and ImageJ (National Institutes of Health, United States) was used to analyze the images.

#### 2.5.2 ARS staining

ARS staining was used to detect calcium mineralization. Cells were cultured in the same manner as ALP staining in a 24-well plate for 7, 10, and 14 d. The osteogenic medium was washed twice with DPBS and fixed with 4% paraformaldehyde (Wako, Japan) at room temperature for 15 min. Cells were then washed thrice with distilled water and 500 μL of 1% of alizarin red (Sigma-Aldrich, United States) was added to each dish, followed by incubation at room temperature for 10 min in a dark place. The stained plates were photographed, the solution was removed, and the plates were washed thrice with distilled water. To quantify the staining, ARS was solubilized in 250 μL of 10% acetic acid (Sigma-Aldrich, United States) (Ryu et al., 2004), and the absorbance at 450 nm was measured using a microplate reader.

#### 2.5.3 Real-time PCR analysis

Total RNA was isolated from hDPSCs with a RiboEx reagent kit (Geneg All, Korea). cDNA synthesis was carried out using a reverse transcription kit (Promega, United States) on 2 µg of total RNA. Real-time PCR quantification was performed using a SYBR Green premix (Applied Biosystems, United States). The oligonucleotide primers for real-time PCR were designed as follows: β-actin: 5′-ACT​CTT​CCA​GCC​TTC​CTT​CC-3′ and 5′-TGT​TGG​CGT​ACA​GGT​CTT​TG-30; ALP: 5′-ATT​TCT​CTT​GGG​CAG​GCA​GAG​AGT-3′ and 5′-ATC​CAG​AAT​GTT​CCA​CGG​AGG​CTT-3′; OCN: 5′GAA​GCC​CAG​CGG​TGC​A-3′ and 5′-CAC​TAC​CTC​GCT​GCC​TCC-3′; OPN: 5′-GCC​AGC​AAC​CGA​AGT​TTT​CAC-3′ and 5′-TGC​ACC​ATT​CAA​CTC​CTC​GC-3′. Amplification conditions consisted of 1 cycle at 95°C for 10 min, followed by amplification for 40 cycles, each at 95°C for 15 s, 60°C for 60 s, and 72°C for 7 s. Subsequently, a melting curve program was applied with continuous fluorescence measurement. Quantitative analysis was performed by ABI 7500 Real-Time System (Applied Biosystems, United States). Each experiment was repeated in triplicate for each individual sample.

### 2.6 Statistical analysis

Quantitative data are presented as the mean ± standard deviation, which was obtained from at least three replicates. A comparison between the two means was made using the student’s independent *t*-test, with statistical significance set at *p* < 0.05.

## 3 Results

### 3.1 Formation of lithium-doped biphasic calcium phosphates

**FIGURE 1:**
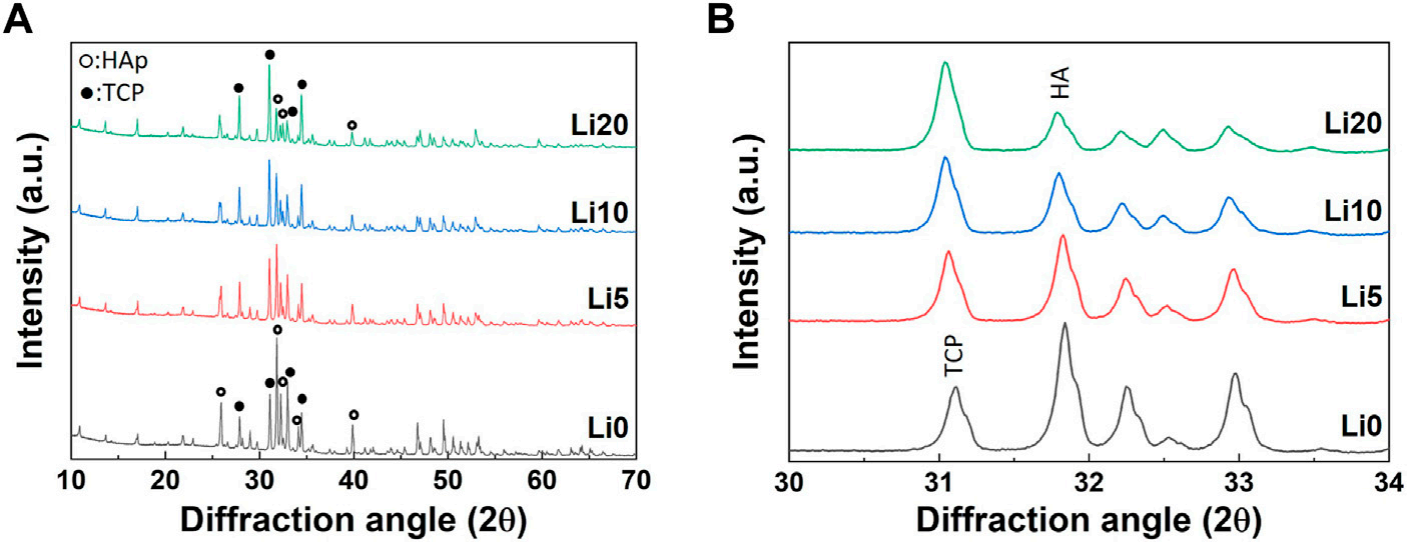
The pure BCP and Li-BCP powders after calcined at 1,000°C; **(A)** XRD patterns and **(B)** magnified XRD patterns for the (2 1 1) and (0 2 10) peak corresponding to HAP and B-TCP phase, respectively.

Li-BCP powders with different doping contents were synthesized using a chemical co-precipitation method. We labeled the synthesized powders as Li 0, Li 5, Li 10, and Li 20 corresponding to pure BCP, 5 at%, 10 at%, and 20 at% Li-doped BCP, respectively (Table 1). The XRD patterns of Li-BCP powders calcined at 1,000°C are shown in Figure 1. As shown in Figure 1A, all obtained powders had both the β-TCP and HA phases, confirming the formation of biphasic calcium phosphates without any other secondary phase. As shown in Figure 1B, the main peak corresponding to the (0 2 10) plane for the β-TCP phase was slightly shifted toward a low 2θ angle with an increase in the amount of lithium ions, whereas the position of the maximum intensity peak for the HA phase did not change with the addition of lithium ions. Additionally, the main peak intensities of (2 1 1) and (0 2 10) corresponding to the HAp and β-TCP phases, respectively, varied with increasing Li content compared to pure BCP. In particular, the peak intensity of the β-TCP phase increased with increasing Li content, whereas that of the HA phase decreased with increasing Li content.

**TABLE 1:** Notation of different content of Li ions substituted in BCP samples.

Sample	Li mol%(calculated)	Li mol%(measured by ICP-AES)
Li0	0	0
Li5	5	4.99
Li10	10	10.00
Li20	20	20.00

**FIGURE 2:**
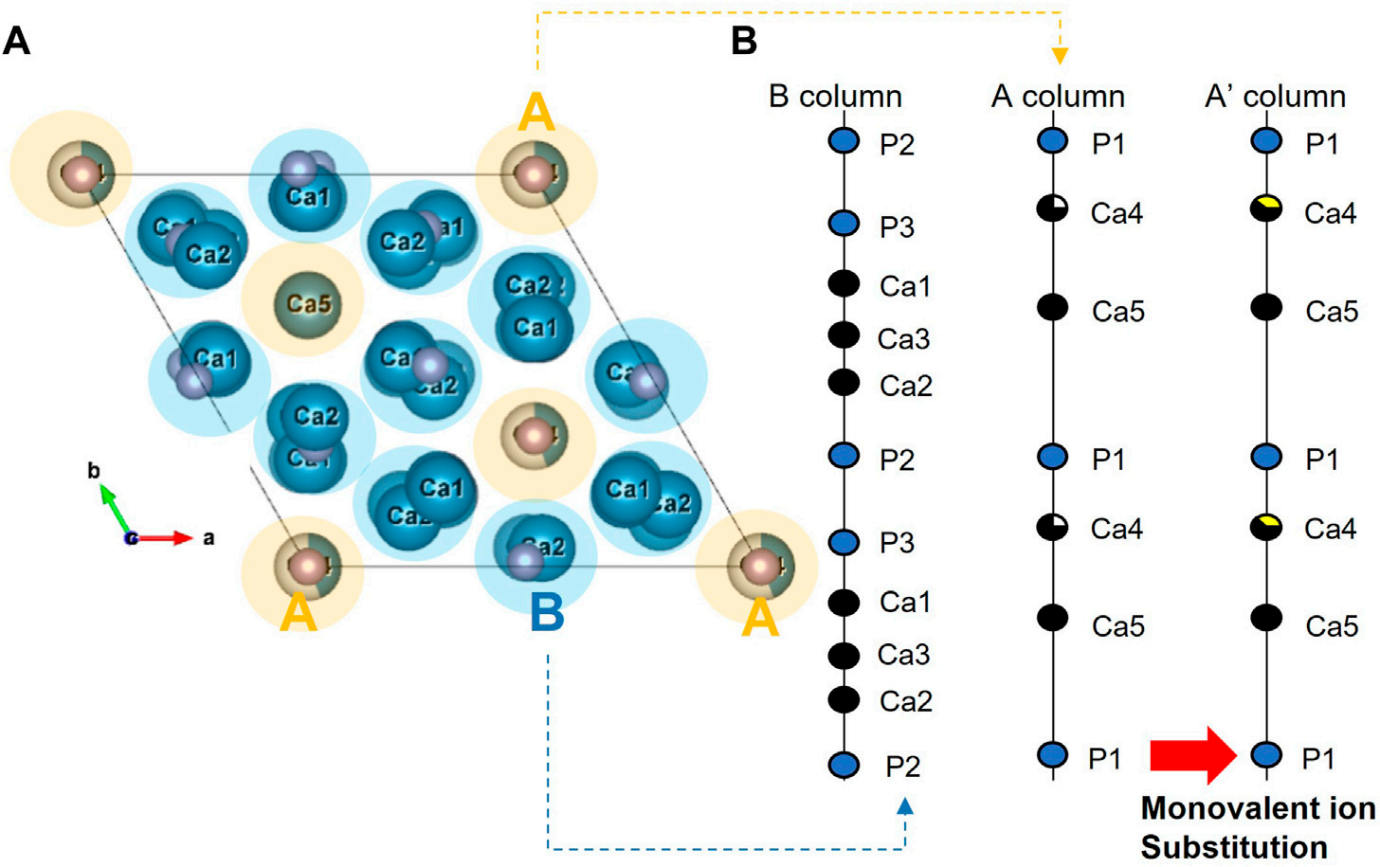
Lattice structure model of B-TCP from the view of **(A)** “c’’ axis and **(B)** along ‘‘c’’ axis.

Considering the variation in the structure of β-TCP with the doping metal ion, it is necessary to evaluate this in more detail. Figure 2 shows the overall crystal structure of β-TCP, consisting of two distinct columns with five non-equivalent Ca sites (Yoshida et al., 2006). As shown in Figure 2, if two lithium ions insert into the Ca (4) site and vacancy site, the A column parallels to the “c’’ axis will be increased with the addition of lithium-ion because of the occupying the vacancy site even though the ionic radius with six-fold coordination of Li^+^ (0.076 nm) is smaller than that of Ca^2+^ (0.100 nm).

**FIGURE 3:**
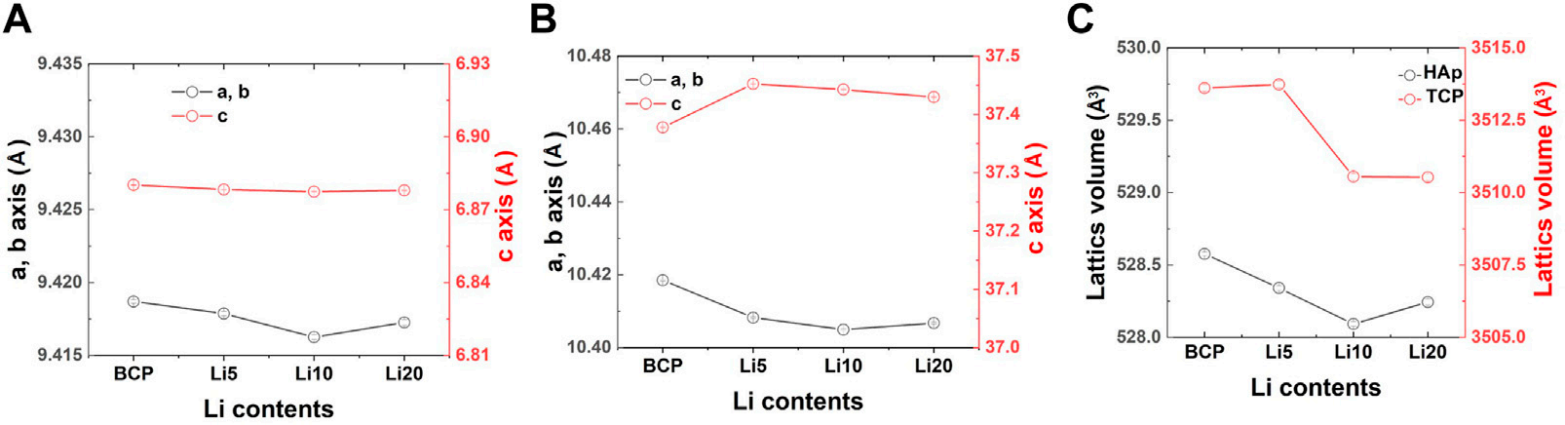
Changes in the lattice constants (a and c axis length, and unit cell volume) of **(A)** HAP, **(B)** B TCP and **(C)** unit cell volume with different lithium contents.

The results after the Rietveld refinement are shown in Supplementary Figure S1, and the reliability factors are listed in Supplementary Table S1. As shown in Supplementary Figure S1, the calculated lines agreed well with the observed lines, and the difference curves (Yobs–Ycalc) were almost linear. The two blue lines represent the Bragg positions for each phase. As shown in Figure 3, the lattice parameters and unit cell volume of HAp did not change significantly with an increase in the amount of lithium. In contrast, the c-axis parameter of β-TCP increased with the addition of lithium (Figure 3B). As shown in Figure 3C, the unit cell volume of β-TCP decreased with increasing amounts of Li.

**FIGURE 4:**
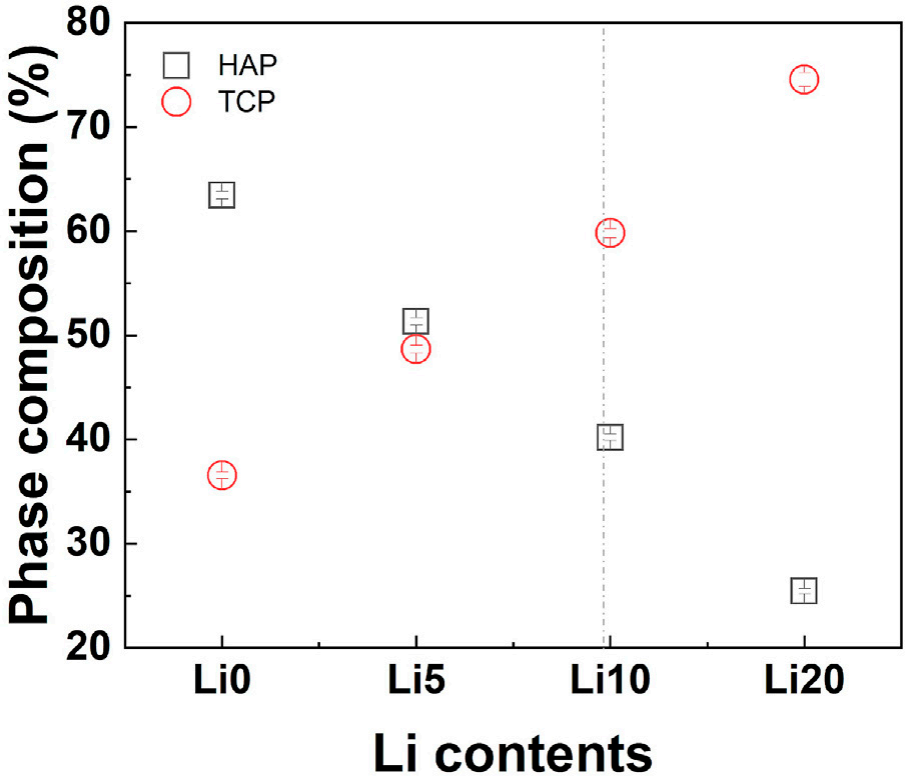
Quantitative phase estimation results from the Rietveld refinement; squares and circles indicate the weight amounts of HAP and B-TCP, respectively. The vertical line for maximum substitution.

The amount of the HA/β-TCP phase in the Li-BCP was also estimated by Rietveld refinement. The results show that the amount of β-TCP phase in Li-BCP increased with increasing lithium content, as shown in Figure 4. As a result, the increase in lithium content reduced the Ca/P ratio for preparing the BCP powder and increasing the amount of the β-TCP phase. However, at higher lithium content of 20 at%, the amount of β-TCP phase was steeply increased to approach 75%. Because substitution occurred in the β-TCP phase, the detailed structural changes were explained by the site occupancy value according to Rietveld refinement (Supplementary Table S2). As mentioned above, among the five independent Ca sites in the β-TCP structure, Li ions substituted the Ca (4) sites and the vacancies near that site. However, when the doping amount of Li ions was higher than 9.09 at%, it could also be substituted into the Ca (1) sites (Morozov et al., 2000). As the lithium content increased, the occupancy of lithium at the Ca (4) sites increased, and the total occupancy of the Ca (4) sites also increased. In particular, for the Li 20 sample, lithium started to simultaneously enter the Ca (1) site with Ca (4) and vacancy [V_Ca_(4)] sites, such as in the formation of Ca_10_Li(PO_4_)_7_ (Morozov et al., 2000).

**FIGURE 5:**
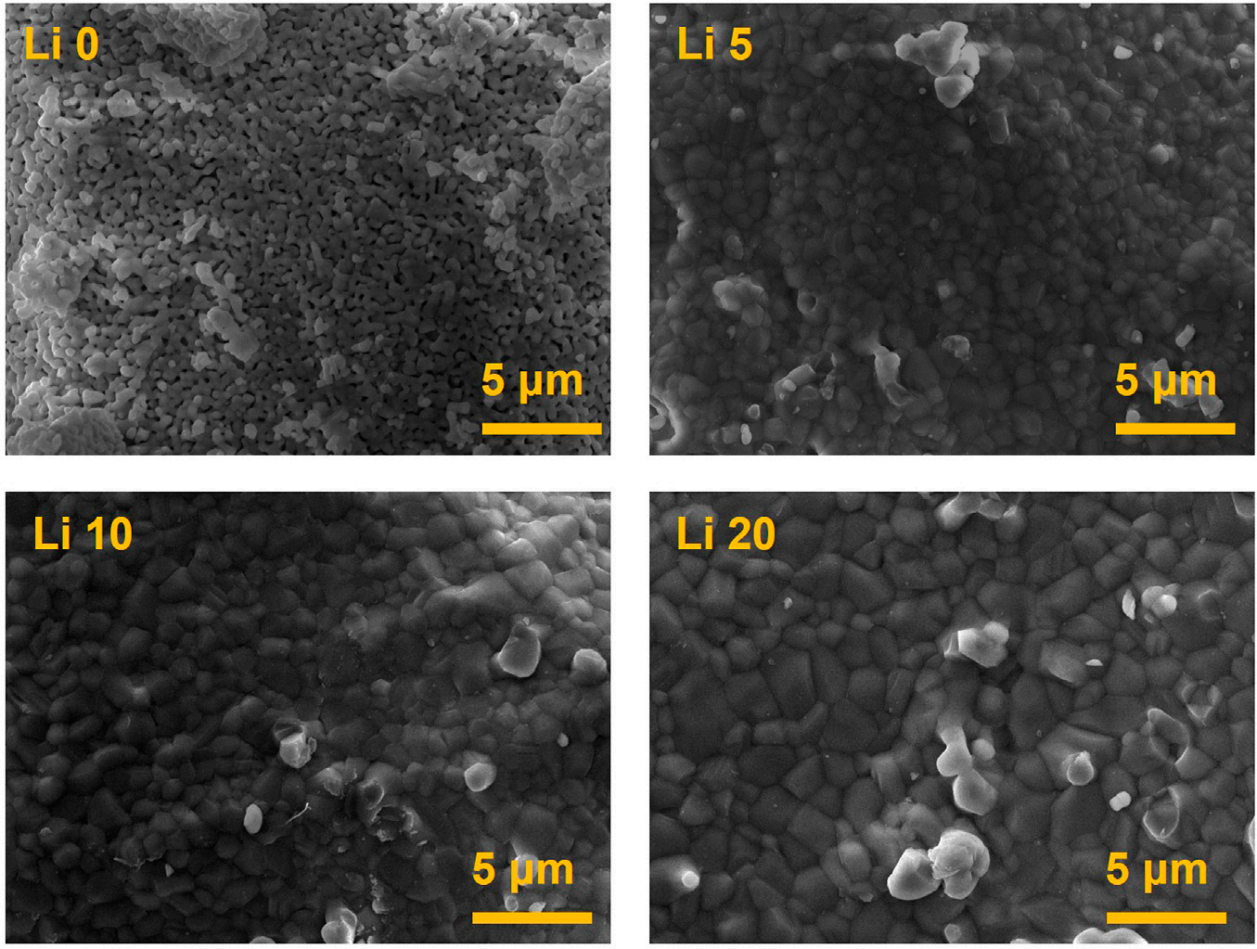
Morphologies of the pure BCP and Li-BCP powders after calcined at 1,000°C.

The surface morphology of the pure BCP and Li-BCP powder calcined at 1,000°C are shown in Figure 5. Li 0 powder show the presence of micropores, however, the lithium-containing powder did not have any pore structures. In addition, the size of grain was increased with the increasing of lithium-ion content.

**FIGURE 6:**
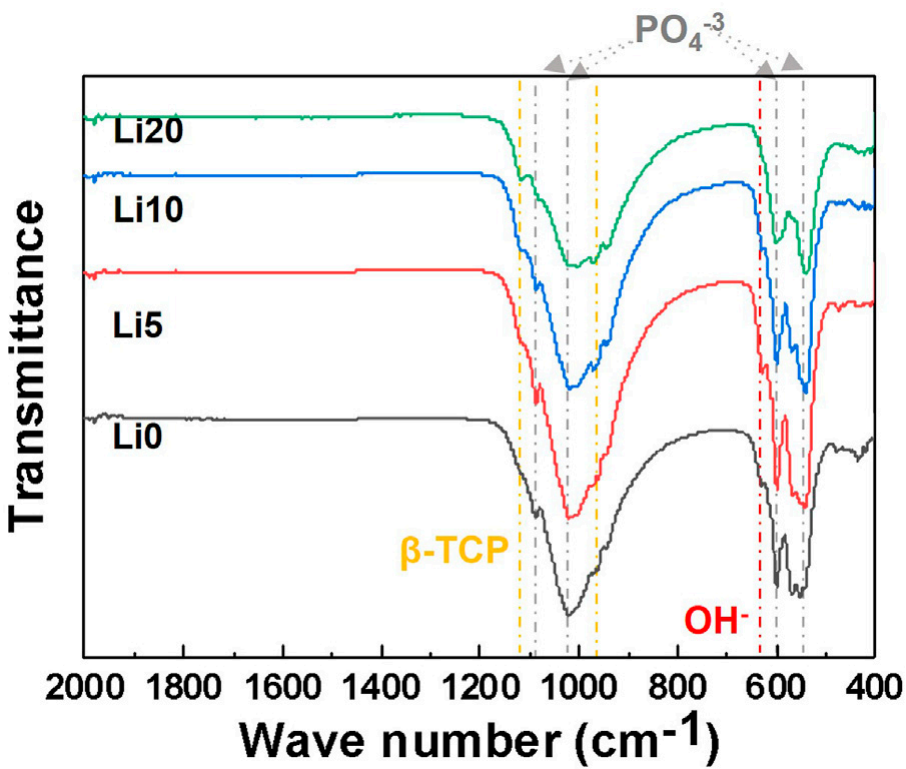
FT-IR spectra of the pure BCP and Li-BCP powders after calcined at 1,000°C.

The FT-IR spectra of calcined powder are shown in Figure 6. The overall spectra appeared mainly in two groups corresponding to PO_4_^−3^ and OH^−^ related to BCP. The transmittance bands at 1990 and 632 cm^−1^ were attributed to the characteristic absorption of OH^−^ (Lu et al., 2021), and the absorption bands at 1,120, 1,020, 603, and 574 cm^−1^ corresponding to the characteristic absorption of PO_4_^−3^ (Suchanek et al., 2004). Among these peaks, two peaks at 1,120 and 974 cm^−1^ are characteristic of β-TCP (Meejoo et al., 2006) and the intensity of these peaks increased with the addition of Li ions. Furthermore, the intensity of the peaks at 632 cm^−1^ assigned to the OH^−^ group (Kim et al., 2012a) decreased, broadening the PO_4_^−3^ bands with lithium doping.

**FIGURE 7:**
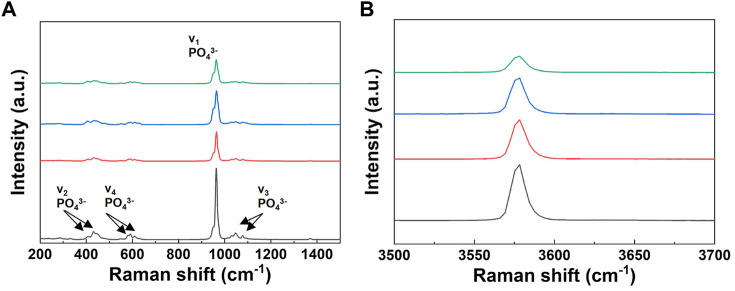
Raman spectra of the pure BCP and Li-BCP powders after calcined at 1,000°C; **(A)** symmetric vibration (stretching mode, v1) of PO34, **(B)** O-H vibration (stretching mode).

The Raman spectra of the powders are shown in Figure 7. As shown in Figure 7A, all spectra indicated four characteristic bands of the PO_3_^−4^ group. The Raman spectra of the powders with increasing lithium content showed a decrease in the intensity of the P-O peak corresponding to the symmetric vibration (stretching mode, ν1) of the HA structure. In addition, to identify the variation in HAp in detail, an enlarged graph including only the OH^−^ band is shown in Figure 7B. The position of this band did not show any difference but only changed its intensity, which decreased with increasing the lithium content.

### 3.2 Cytotoxicity and proliferation

**FIGURE 8:**
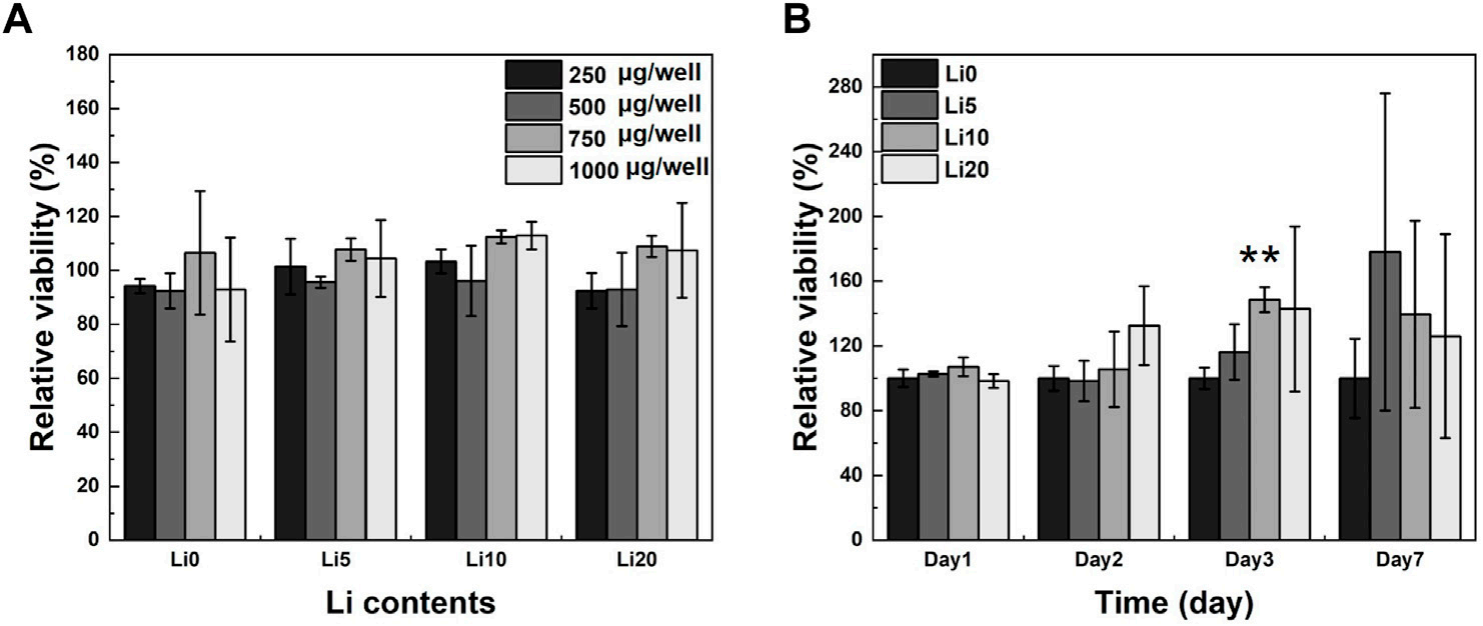
hDPSCs optical cell density on pure BCP and Li-BCP powders; **(A)** with different powder concentration for 1 day, and **(B)** with different culture times at 1,000 µg/well (vs. control). Asterisks indicate statistical significance (**p < 0.01) with respect to pure BCP at the same time point as determined by independent *t*-test.

MTT assay was used to determine the cytotoxicity and proliferation of human dental pulp stem cells (hDPSCs). To determine the proper concentration of the powder, the MTT assay was performed with different powder concentrations. As shown in Figure 8A, none of the Li-BCP powders showed similar values to control (with full media), which had no cytotoxic effects on hDPSCs regardless of powder concentration. In general, cytotoxicity property would be favored with increasing powder concentration. Therefore, the following osteogenic cell behavior test was conducted at a powder concentration of 1 mg/well. The proliferation of hDPSCs cultured in Li-BCP powders for 1, 2, 3, and 7 d is shown in Figure 8B. The relative cell viability was statistically higher for all lithium-doped powders than for pure BCP powder.

### 3.3 Extraction of Li-BCP

The amounts of Ca, P, and Li released in the α-MEM solution in 1 day are presented in Table 2. As shown in Table 2, the concentration of lithium ions in the extracts steadily increased up to 10 at% lithium doping. However, in the case of the Li 20 sample, the amount of extracted lithium was drastically increased compared to the Li 5 and Li 10 samples. All the extracts had higher concentrations than the control specimens for the release of Ca and P ions. The released amount of Ca was the highest in the Li 10 sample, while the released amount of P was the highest in the Li 20 sample. The overall degradation behavior of the Li-BCP samples increased with increasing Li content. To understand the variation in the crystallinity and chemical state of Li-BCP samples after immersion in α-MEM solution for 1 day, the Li-BCP samples were characterized using XRD, FT-IR, and Raman spectroscopy. The XRD patterns and chemical states of the extracted Li-BCP samples are shown in Supplementary Figure S2. The crystallinity of the extracted Li-BCP samples slightly decreased, as shown in Supplementary Figure S2A. For the Li 5 and Li 10 samples, the variation in crystallinity was lower than that of the Li 0 and Li 20 samples.

**TABLE 2:** The ion concentration in supernatant after the Li-BCP powders with different lithium contents soaked in a-MEM solution for 1 day (control: a-MEM).

mmol/L	Li	Ca	P
Li0	0	1.438	1.045
Li5	0.049	1.495	1.141
Li10	0.081	1.549	1.148
Li20	1.418	1.468	1.159
ctrl		1.422	0.890

### 3.4 Osteo/odontogenic differentiation behavior in vitro

**FIGURE 9:**
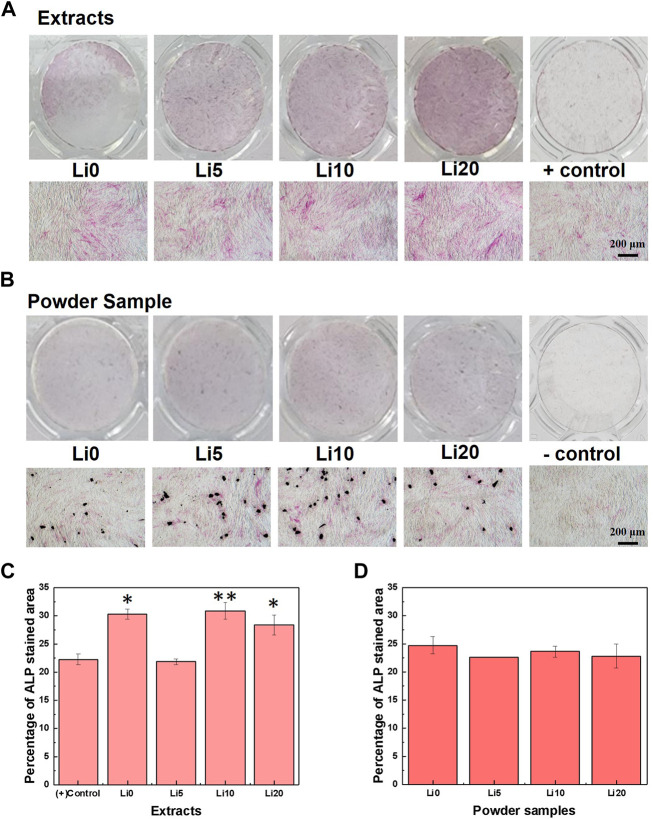
ALP staining results after 7 days; **(A)** 0.1 g/ml extracted samples and **(B)** 1mg/well powder samples, respectively, and quantified results of **(C)** 0.1 g/ml extracted samples and **(D)** 1mg/well powder samples. Asterisks indicate statistical significance (*p < 0.05 and **p < 0.01) with respect to positive control as determined by independent *t*-test.

The effect of lithium ions on the osteo/odontogenic differentiation of hDPSCs was evaluated using ALP an early marker and mineralization a late marker. The ALP staining results of hDPSCs cultured in the extracts after 7 days are shown in Figure 9. In the case of extracts, the red staining was to become denser with increasing the amount of lithium as shown in Figure 9A. However, as you can see in Figure 9B, the red staining for the powder samples was not much change with the amount of lithium. At Li 5, Li 10, and Li 20, the staining became stronger around the particles (black area). Meanwhile, for more understanding of the ALP staining results, the stained area fraction was carried out using ImageJ and the results are shown in Figures 9C,D. In the case of extracts (Figure 9C), all extracts except Li 5 shows the higher value than positive control. Among them, Li 0 and Li 10 extracts have highest fraction of differentiation. On the other hand, there was no statistically significant difference between positive control and powder sample as shown in Figure 9D.

**FIGURE 10:**
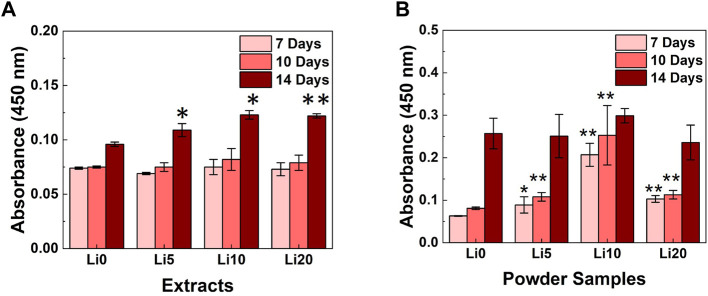
ARS activity results for the different culture times; **(A)** 1 mg/well powder samples and **(B)** 0.1 g/ml extracted samples. Asterisks indicate statistical significance (*p < 0.05, **p < 0.01) with respect to Li 0 at the same time point as determined by independent *t*-test.

To evaluate the degree of mineralization, ARS activity was performed with the extracts and powder samples and the results shown in Figure 10. As for the extracts (Figure 10A), there were not statistically differences in staining were observed until 10 days. After 10 days, the increase in staining was observed for Li- BCP (Li 5, Li 10, and Li 20) compared with the Li 0 extracts. And the absorbance value increased slightly with increasing during culture time, and Li 10 and Li 20 extracts exhibited maximum absorbance values at day 14. In Figure 10B, the Li-BCP powder samples show a statically significant increasement compared to Li 0 powder from 7 days. At each time, the mineralization potential was promoted with increasing the lithium contents, and Li 10 powder have highest value than other sample.

**FIGURE 11:**
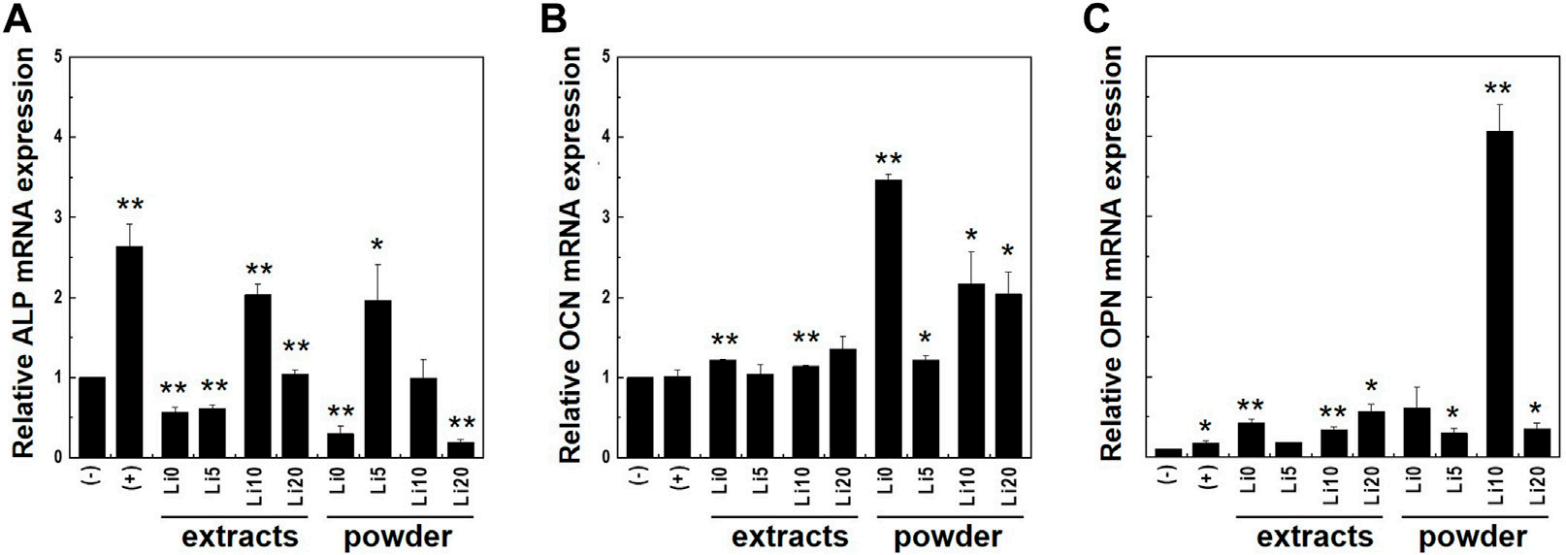
Expression levels of osteo/odontogenic differentiation markers; **(A)** ALP and **(B)** OCN, and **(C)** OPN were analyzed with real-time PCR. Cell were cultured both in full medium [(-)control] and ODM [(+)control]. Asterisks indicate statistical significance (*p < 0.05, **p < 0.01) with respect to (-)control at the same time point as determined by independent *t*-test.

To further evaluate osteo/odontogenic differentiation, quantitative real-time PCR were performed with osteo/odontogenic markers: ALP, osteocalcin (OCN), and osteopontin (OPN) on days 7. These genes were normalized with β-actin and results were shown in Figure 11. As shown in Figure 11A, the mRNA expression of ALP had the highest value at control with ODM [(+) control] compared to powder and extracts samples. Meanwhile, in the case of powder samples, higher concentrations of lithium (Li 10 and Li 20) show higher values than low concentrations (Li 0 and Li 5). However, for the extracts, expression levels were increased at Li 5, and Li 0 and Li 20 show lowest value.

For the OCN, the mRNA expression was enhanced in all samples compared to that in both control (Figure 11B) and the powder samples had higher value overall. Both extracts and powder sample of Li 5 show lower value than other samples. In Figure 11C, the mRNA expression levels of OPN increased compared to that of controls. In the case of powder sample, Li 10 shows significantly higher value than other samples.

## 4 Discussion

Doping calcium phosphate ceramics with various trace elements are used to enhance their properties (mechanical, chemical, and thermal) for clinical applications (Gozalian et al., 2011; Sprio et al., 2020; Yoo et al., 2020). However, it is difficult to define the actual effects of the doping ion itself because it reinforces chemical bonding or decreases material stability. Therefore, when ion-doped calcium phosphates show better biological properties, it is necessary to analyze whether this effect is caused by the released ions or surface stability. To stimulate biological processes, the release of these trace ions is important (Barralet et al., 2009). However, to increase the adsorption of cells and proteins, a stable surface is required (Samavedi et al., 2013). In this study, we expected that lithium ions would improve the biological behavior of BCP by stimulating the osteogenic pathway. Thus, we focused on the release of lithium from the base materials and selected the amount of lithium ions that exceeded the substitution limit.

The structural analysis presents the substitution site in BCP, and the phase stability related to its degradation behavior. First, the XRD results indicate that Li-BCP powders with different lithium contents were successfully prepared. According to previous studies, the main peak of lithium-doped HA show shifts slightly toward right compared to HA (Padmanabhan et al., 2019) and lithium-doped β-TCP rarely changed in the XRD graph (Matsumoto et al., 2010). In this study, the main peak of the β-TCP phase moved toward a lower angle owing to the increase in the crystal space with the addition of lithium content (Matsumoto et al., 2010). Therefore, the substitution of lithium would be preferred to enter in β -TCP phase of BCP. Second, the results of the Rietveld refinement indicated that lithium was preferentially incorporated into β-TCP, which is consistent with earlier reports (Kannan et al., 2005; Lu et al., 2021). According to Yoshida et al. (Yoshida et al., 2006), monovalent atoms can substitute Ca (4) sites, including V_Ca_(4), up to a substitution content of 9.09 at% as follows:

$$2{Li}^{+}={Ca}^{2+}+V_{Ca\left(4\right)}$$

When lithium ions were inserted into β-TCP, the Li + ions occupying the Ca (4) sites and vacancies were surrounded by three oxygen atoms, giving a coordination number (CN) of 3. These Li-related areas are larger than the original Ca sites (Morozov et al., 2000). However, the lattice volume decreased with Li doping, indicating an improvement in the crystal stabilization. This stabilization is caused by the insertion of Ca^2+^ and Li^+^ into all Ca sites and vacancies in the β-TCP structure (Matsumoto et al., 2010). In terms of the phase composition in BCP, the changes are also related to lithium ions. In detail, Li-BCP powders with Ca/P ratios lower than 1.602 lacked calcium ions, revealing a reversion of the composition (Li 10 and Li 20 samples) with the addition of lithium ions. The reversion was observed to be nearly 10 at%, which could be due to excess lithium substitution into calcium. In the SEM images, the lithium ions effect on the densification behavior and did not show the liquid phase like Li-TCP (Vahabzadeh et al., 2017). This densification was like magnesium doped BCP (Hanif et al., 2020), therefore, lithium ion could affect the sinterbility of BCP.

Finally, the results of the chemical bonding also indicate the doping sites of lithium ions. In the FT-IR spectra, when the lithium-ion was doped in BCP, the bands related to β-TCP were more intense, with broadening of the OH- bands. On the other hand, it was not found that the absorption peak at 875 cm^−1^ was related to HPO_4_^−2^ groups. These tendencies are typical for ion-substituted calcium phosphates synthesized by wet methods (Suchanek et al., 2004), and it should also be considered that lithium ions can be preferentially inserted into the β-TCP phase. The broadening of the bands was associated with the increased lattice disorder, and this instability increased with the lithium content. The Raman spectra of the powders with increasing lithium content also showed a decrease in the intensity of the P-O peak of the HAp structure. This could be due to the reduction in the HAp phase with the addition of lithium ions. These results suggest that the lithium-ion is favored as a substitute for the Ca site in the β-TCP phase in the BCP structure, which is in good agreement with other ion-doped BCP-related previous reports (Lu et al., 2021). Combining the above three analysis results, the lithium-ion doped in the β-TCP phase makes dissolution easier, whereas it stabilizes the β-TCP crystal in BCP.

The release of lithium ions was investigated using an extraction method (Standardization, 2012), and the extraction time (1 day) is reasonable for comparing the BCP and Li-BCP series (Matsumoto et al., 2010). Quantitative results revealed that all powders started to degrade during 1 day in the α-MEM solution. Accelerating the degradation of Li-BCP with lithium ions is related to the decrease in the Ca/P ratio. This could also be attributed to crystal instability due to the excess substitution amount, as mentioned above. However, unlike our structural conception, Ca ions and P ions are also released much more in the Li 10 sample. Therefore, the structural stability decreases during degradation. In addition, the chemical state of Li-BCP powder did not change significantly after immersion.

The biological behavior of Li-BCP was investigated *via* cytotoxicity, proliferation, and differentiation tests using hDPSCs. Both pure BCP and Li-BCP exhibited no toxicity, and Li 10 showed good proliferation behavior. The osteogenic differentiation behavior was measured by staining methods using both early and late marker. Also, these biological properties identifying the effect of initial released products by using extracts, and effect of materials during experimental period by using powder samples. In the ALP staining results, only for the extracts show the increasing of stained area compared to positive control. However, this increment was regardless of releasing lithium-ion. Mineralization was detected more in the Li 10 sample than other sample in both extracts and powder samples. As for the Li 20 sample, it did not show the improvement of mineralization behavior compared to Li 10 powder. Also, Li 20 extracts even show the decrease of absorbance compared to Li 10 extracts. These phenomena could be affected by the concentration of released ion. Li 20 extracts contained the highest concentration of lithium, and the calcium ion concentration is lower than Li 5 extracts. Therefore, this high concentration of lithium ions would have cytotoxicity and cause to negative effects on the biological properties (Timmer and Sands, 1999; Young, 2009).

Further osteogenic differentiation of hDPSCs was investigated with the expression of related markers. ALP is crucial for extracellular matrix (ECM) mineralization (Bou Assaf et al., 2019). Also, OCN promotes the deposition of minerals in ECM and OPN regulates bone formation and mineralization (Amarasekara et al., 2021). For extracts, Li 5 sample has lowest value and expression level increased at Li 10 and Li 20. It is same tendency to ALP staining and these results present the inducing of osteogenic differentiation at Li 10 and Li 20 extracts. According to enhancement at Li 20 extracts rather than Li 0 extracts, released lithium ions effect on the osteogenic behavior of hDPSCs. However, at the lower concentration below 0.08 mM (Li 5), released lithium ion did not improve the osteogenic properties. For the powder sample, the tendency of expression levels was different from kinds of gene. The ALP expressions were activated at Li 5 and Li 10, and the enhancement of OCN and OPN was revealed at only Li 10 powder. The lower ALP expression is related with the differentiation process. When the mature osteoblasts were synthesized, ALP expression level was decreased (Zhou et al., 2021). Therefore, Li 10 powder, having higher expression level both OCN and OPN, would be activated osteo/odontogenic differentiation process. Based on the results of biological test, Li 10 sample show highest potential of osteo/odontogenic differentiation.

## 5 Conclusion

In this study, pure BCP and Li-substituted BCP with different contents were fabricated using the co-precipitation method. Lithium preferentially entered the β-tricalcium phosphate phase by substituting Ca (4) and calcium vacancy sites up to a lithium content of 10 at%. Lithium substitution improved the crystal stabilization by the formation of a large space around Li ions and inducing the release of Li + at the degradation test for 1 day. Also, the lithium released from Li-BCP revealed improvement of osteogenic differentiation of human dental pulp stem cells. However, at higher than 10 at%, the excess amounts of lithium did not give additional enhancement. Based on our experimental results, the most effective lithium doping content in BCP for improving its osteo/odontogenic potential is approximately 10 at%. In addition, our results highlight the importance of analyzing material structures and determining their relationship with biological properties. After further validation, our findings can be used in clinical settings to treat bone defects.

## Data Availability

The original contributions presented in the study are included in the article/Supplementary Materials, further inquiries can be directed to the corresponding author.
