# Supplementary material for: Lithium doped biphasic calcium phosphate: Structural analysis and osteo/odontogenic potential in vitro
Source: Front Bioeng Biotechnol. 2022 Nov 8;10:993126. doi: 10.3389/fbioe.2022.993126 (PMC9679216; doi:10.3389/fbioe.2022.993126)
Supplement: Supplementary file 1 [file DataSheet1.PDF]

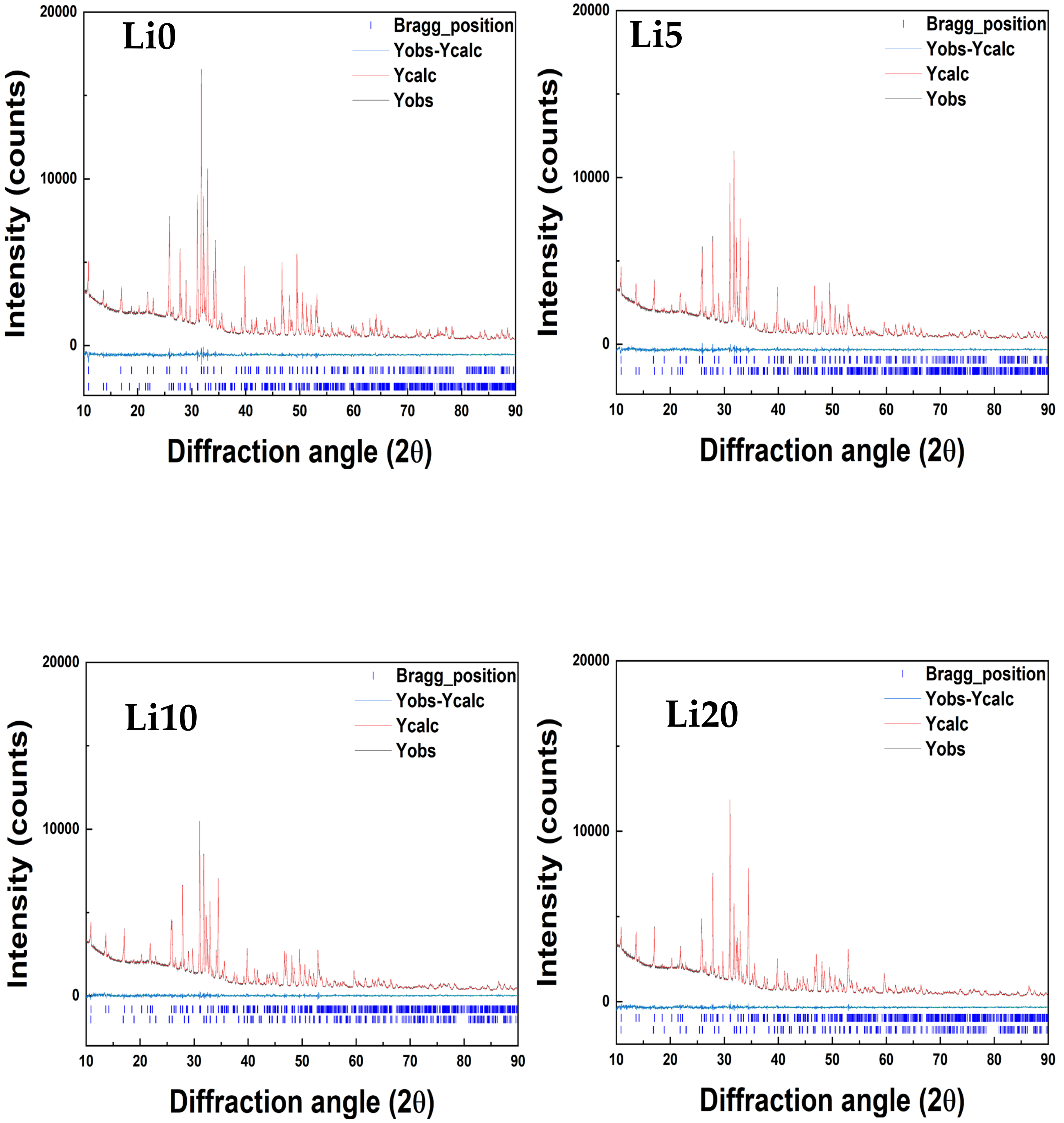

Fig. S1. Rietveld refinement results

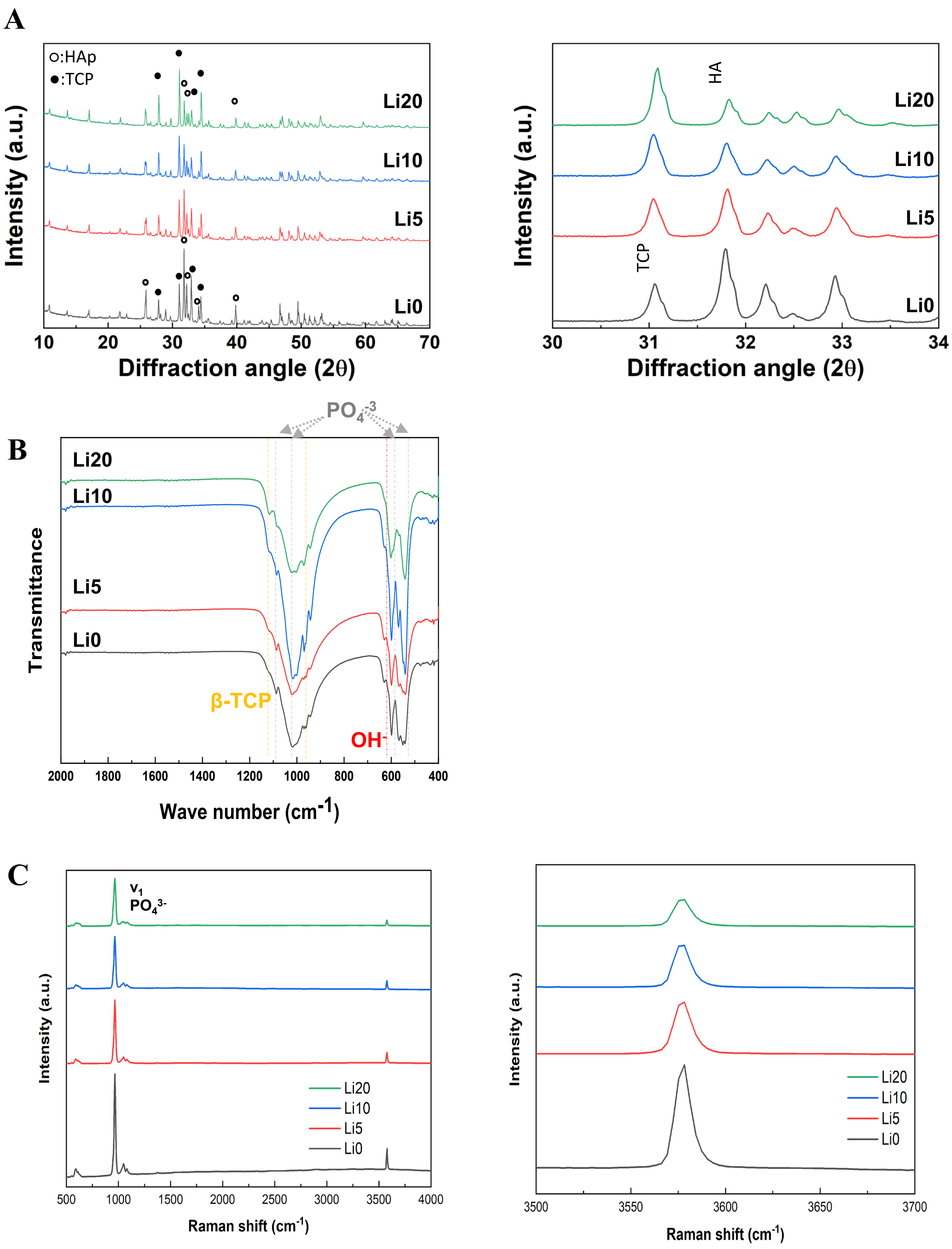

Figure S2. (A) XRD patterns, (B) FTIR spectra and (C) Raman spectra for the Li-BCP samples extracted after immersion in α-MEM solution for 1 day, respectively.

Table S1. The reliability factors of Li-BCP in the Rietveld refinememt

**Sample**	**Li (mol%)**	**Reliability factors**	**Bragg R-factor factors**
		**$R_p/R_{wp}/R_{exp}$/Chi2**	**HAp/TCP**
**Li0**	0	3.33/4.31/3.30/1.70	4.98/ 7.75
**Li5**	5	2.59/3.39/2.86/1.40	2.67/3.69
**Li10**	10	2.49/3.26/2.87/1.29	2.71/3.19
**Li20**	20	2.55/3.33/2.89/1.33	2.68/3.48

Table S2. The occupancy of lithium ion in β-TCP structure with different lithium contents

**Site occupancy**	**Ca(1)**		**Ca(4)**	
	**Ca**	**Li**	**Ca**	**Li**
Li0	1	-	0.692(17)	-
Li5	1	-	0.425(14)	0.469(14)
Li10	1	-	0.339(13)	0.661(13)
Li20	0.953(11)	0.047(11)	0.358(9)	0.642(9)
